# Supplementary material for: The Expression Kinetics and Immunogenicity of Lipid Nanoparticles Delivering Plasmid DNA and mRNA in Mice
Source: Vaccines (Basel). 2023 Oct 11;11(10):1580. doi: 10.3390/vaccines11101580 (PMC10610642; doi:10.3390/vaccines11101580)
Supplement: Supplementary file 1 [file vaccines-11-01580-s001.zip › vaccines-2561858-SI.pdf]

<u>**Supplementary Materials**</u>

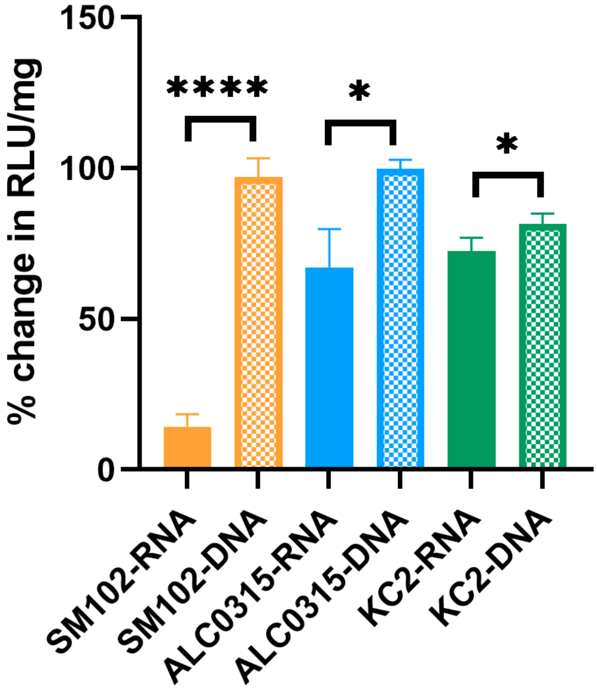

Figure S1. DNA-LNPs are more thermostable than RNA-LNPs. LNPs encapsulating mRNA encoding firefly luciferase were stored at 37°C for seven days and then used to transfect HEK293T cells according to the *in vitro* transfection assay protocol described in the methods section. % change in RLU/mg represents the change in RLU/mg when compared to that of the freshly prepared LNP formulation on day 0. RLU: relative luminescence units. Error bars represent standard deviation (SD). * p-value < 0.05, **** < 0.0001.
